# Isolation and Optimisation of Culture Conditions for a Marine Bioflocculant-Producing Bacterium and Application of Its Bioflocculant in Wastewater Treatment

**DOI:** 10.3390/ijerph191610237

**Published:** 2022-08-18

**Authors:** Tlou Nelson Selepe, Tsolanku Sidney Maliehe, Kgabo Moganedi, Peter Masoko, Vusimuzi Mulaudzi

**Affiliations:** 1 Department of Water and Sanitation, University of Limpopo, Private Bag X1106, Polokwane 0727, South Africa; 2 Department of Biochemistry, Microbiology and Biotechnology, University of Limpopo, Private Bag X1106, Polokwane 0727, South Africa; 3 Department of Chemistry, University of Limpopo, Private Bag X1106, Polokwane 0727, South Africa

**Keywords:** *Ochrobactrum oryzae* AB84113, bioflocculant, flocculating activity, removal efficient, wastewater

## Abstract

The application of bioflocculants has become an alternative to that of chemical flocculants in wastewater treatment due to their environmental friendliness and non-toxic effects. This study aimed at isolating a bioflocculant-producing bacterium from marine water, optimisation of its culture conditions, and investigation of the removal efficiency of its bioflocculant on pollutants in wastewater. The bacterium was identified by 16S rRNA gene analysis. Optimal carbon and nitrogen sources, inoculum size, temperature, pH, and time were determined by the one-factor-at-a-time assay. The cytotoxicity of the bioflocculant was assessed on African green monkey kidney and bovine dermis cells using a tetrazolium-based columetric (MTT) method. Its removal efficiencies on chemical oxygen demand (COD), biological oxygen demand (BOD) and sulphur were determined using the Jar test method. The bacterial isolate was identified as *Ochrobactrum oryzae* AB84113. A maximum flocculating activity of 92% and a yield of 3.768 g/L were obtained when a 1% (*v*/*v*) inoculum size was used in the presence of starch and yeast extract at pH 7, 30 °C, and after 72 h of cultivation. The bioflocculant demonstrated non-cytotoxic effects on bovine dermis and African green monkey kidney cells. The bioflocculant removed 98% COD, 91% BOD and 86% of Sulphur. The bioflocculant has potential for pollutant removal from industrial wastewater.

## 1. Introduction

Water is an essential resource for life. However, water quality is constantly deteriorating due to increased contamination from industrial activities and urbanisation [1]. Globally, approximately 80% of wastewater is not properly treated prior to its discharge into various water bodies [2]. Water pollution affects more than 1.2 billion people globally, leading to poor economic growth and food insecurity [3]. Moreover, this leads to a challenge in achieving environmental sustainability as addressed by the Millennium Development goal No.7 [4]. Thus, it is it imperative to effectively treat wastewater and water to appraise its quality.

Flocculation is a process employed in solid-liquid separation in several industrial processes such as wastewater treatment, drinking water purification, food and fermentation processes [5]. Flocculants are agents that reduce or eliminate the stability of colloids in suspensions, enabling the dispersed particles to aggregate. Generally, flocculants are grouped into inorganic (i.e., aluminium sulfate, aluminium chloride, ferric chloride and ferrous sulphate), organic synthetic flocculants (i.e., polyacrylamide derivatives and polyethyleneimine) and naturally occurring flocculants (i.e., plant based and microbial flocculants) [6]. Among the three types of flocculants, inorganic and organic synthetic flocculants are mainly used in wastewater treatment because of their availability at low cost and their high flocculating abilities [7,8]. However, these types of flocculants are harmful to the environment and pose health problems [9]. For instance, ferric salts tend to be corrosive due to their hydrolysis, which leads to acidic conditions in water [10]. Aluminium salts are also linked to Alzheimer’s disease in humans [11]. Moreover, polyacrylamide derivatives are often non-degradable in nature and exhibit a resilient biological toxicity [12]. Therefore, alternative eco-friendly and innocuous flocculants are needed.

Natural occurring flocculants such as plant-based and microbial flocculants are the preferred alternatives to inorganic and organic synthetic flocculants [13,14]. Plant-based and microbial flocculants are environmentally friendly and have low or non-toxic effects [15]. Although plant-based flocculants exhibit strong activities in wastewater treatment [14,16], generally their biosynthesis is not convenient in terms of quality and productivity due to ecological conditions and climatic change. Thus, microbial flocculants are gaining more attention because they are quickly reproducible within a short time and are weather-independent [17]. Microbial flocculants are produced by bacteria and fungi and are mainly composed of carbohydrates, proteins, and lipids [18]. Although bioflocculants are promising alternatives, the high costs of their production and the generally low yields are major bottlenecks for their industrial utilisation [15]. Thus efforts to reduce the cost of production and application include screening of high-yield producing microorganisms and optimization of their culture conditions [8].

Several bioflocculant-producing bacterial species have been reported for their significant flocculating activities (>70–90%), yield, and various additional functions in wastewater treatment [19,20]. Much research has been done on bioflocculant-producing bacteria in activated sludge and soil [21,22], but not on those from marine environments [23].

This study aimed at isolating and identifying a marine bioflocculant-producing bacterium from Mtuzini Beach in Kwa-Zulu Natal, South Africa. To maximize bioflocculant yield by the isolate, the medium composition (carbon and nitrogen sources) and culture conditions (inoculum size, temperature, initial pH of the medium and cultivation time) were optimised using the one-factor-at-a-time method. The surface morphology, functional groups, and degradation property of the bioflocculant were identified using scanning electron microscope (SEM), Fourier-transform infrared (FT-IR) spectroscopy and thermogravimetric analysis (TGA), respectively. The biosafety of the bioflocculant was assessed by evaluating its cytotoxicity on the African green monkey kidney and Bovine dermis using a tetrazolium-based columetric (MTT) method. Lastly, its ability to remove materials expressed by biological oxygen demand and chemical oxygen demand, as well as sulphur from wastewater, were determined using the Jar test method.

## 2. Materials and Methods

### 2.1. Chemicals and Production Medium

All chemicals, reagents and media used were of analytical grade and were procured from Merck Pty Limited and Sigma-Aldrich, Johannesburg, South Africa. The selective media used for isolation were marine agar (MA) and reasoner’s 2A agar (R2A). The enrichment medium was composed of 3 g beef extract, 10 g tryptone and 5 g sodium chloride. The production medium by Zhang et al. (2007) was used for evaluation of bioflocculant production. The medium was composed of glucose (20.0 g), KH_2_PO_4_ (2.0 g), K_2_HPO_4_ (5.0 g), (NH_4_)2SO_4_ (0.2 g), NaCl (0.1 g), CH_4_N_2_O (0.5 g), MgSO_4_ (0.2 g) and yeast extract (0.5 g). All media were prepared in filtered marine water before been sterilised by autoclaving at 121 °C for 15 min. 

### 2.2. Sample Collection

Water samples were collected from Mtunzini Beach in KwaZulu Natal, South Africa (28°57′ S 31°45′ E). Sterile Scott bottles were used to collect water samples on three different sites and on each site water samples were collected in triplicate. The physiochemical parameters, temperature, pH, total dissolved solid, dissolved oxygen, salinity and pressure were determined in-situ using HI 98194 PH/E/DO multi-parameters. Thereafter, the water samples were put in an ice box and transported to the laboratory at the Department of Water and Sanitation at the University of Limpopo, South Africa.

### 2.3. Isolation of the Bioflocculant-Producing Bacteria

Two millilitres of the marine water samples were transferred into 8 mL of sterile saline water (0.85% (*w*/*v*)) and agitated for 30 s. Thereafter, serial dilutions were performed and 100 µL of the diluted and undiluted samples were spread on the surface of marine and Reasoner’s 2A (R2A) agar plates. The agars were previously prepared using filtered marine water from Mtunzini Beach and were adjusted to pH 6.8 prior to autoclaving at 115 °C for 15 min. The R2A plates were incubated for 4 days at 27 °C and the marine agar plates were incubated for 8 days at the same temperature. Colonies from both media were selected according to their appearance and morphology. The selected colonies were subculture twice on their isolated agar media.

### 2.4. Activation of the Isolates

Enrichment medium (see Section 2.1) was used to activate bacteria. About 50 mL of the medium was poured into different test tubes and autoclaved at 121 °C for 15 min. A loopful of the isolates was inoculated into the activation broth and incubated in a rotary shaker at 160 rpm, 37 °C for 24 h [24].

### 2.5. Cultivation Medium for Bioflocculant Production

Activated isolates (1 mL) were inoculated into the sterile production medium (see Section 2.1) at 27 °C for 72 h at pH 6.8 and a shaking speed of 160 rpm. After the incubation period, 2 mL of the broth cultures were withdrawn and centrifuged at 10,000 rpm for 15 min. The supernatants were used for the evaluation of flocculating activity.

### 2.6. Determination of Flocculating Activity of Isolates

Cell-free supernatants were used to determine flocculating activity according to Kurane et al. [25]. Briefly, 2 mL of the supernatants and 3 mL of 1% (*w*/*v*) CaCl_2_ were added to 100 mL of kaolin suspension (4.0 g/L, pH 7.0) in a conical flask. The mixture was shaken for a minute, poured into a measuring cylinder and allowed to stand for 5 min. Afterwards, 2 mL of the clarifying upper phase layers were carefully withdrawn and their optical densities (OD) were measured at 550 nm with a spectrophotometer (Spectro-quant, Pharo 300 Merck, Boston, MA, USA). Sterile distilled water (2 mL) was used as the control. The percentage flocculation activity (%FA) of all the supernatants was calculated according to the equation:

%FA = (A_o_ − A)/A_o_ × 100, (1)

where A_o_ and A are the optical densities of the control and the test samples at 550 nm. The bacterial isolate with the highest flocculating activity was selected for identification.

### 2.7. Identification of the Bacterium

The selected bacterial isolate was identified using 16S rRNA gene sequence analysis. Genomic DNA extraction, PCR-mediated amplification of the 16S rRNA gene fragments and sequencing of PCR products were carried out as described by Shida et al. [26]. The obtained sequences were analysed by comparing them with those retrieved from the National Center for Biotechnology Information (NCBI) databases to identify the closest bacterial species. 

### 2.8. Optimization of the Culture Conditions

Different inoculum sizes were used to determine their effect on bioflocculant production. Flasks (100 mL) containing 50 mL of the sterile production medium were inoculated with 0.5, 1.0, 1.5 and 2 mL of the cultured broth to give 1, 2, 3 and 4% (*v*/*v*) inoculum sizes, respectively. The inoculums were then incubated for 72 h at 30 °C at a shaking speed of 160 rpm. The broth cultures were then centrifuged at 10,000 rpm for 15 min and the supernatants were analysed for flocculating activity as described previously [27]. The effect of carbon and nitrogen substrates was determined using the method of Luo et al. [28]. Glucose in the original medium was substituted with 20 g/L of the following carbon sources: fructose, sucrose, maltose, lactose, xylose, starch and molasses. Thereafter, the flocculating activity was assessed as previously stated [29]. Mixed nitrogen sources (urea, yeast extract and (NH_4_)_2_SO_4_) in the original production medium were also replaced with 1.2 g/L of organic (casein, peptone, urea and yeast extract) and inorganic (ammonia) nitrogen sources to determine their effect on bioflocculant production [30]. The flocculating activity was then measured as described previously. The effect of pH of the growth medium was determined according to the method of He et al. [31]. The pH of the medium was adjusted in the range of 3 to 12 using 0.1 M HCl and 0.1 M NaOH and autoclaved. Thereafter, the isolate was inoculated and incubated for 72 h in a rotary shaker at 30 °C and 160 rpm. Flocculating activity was determined thereafter. To assess the effect of temperature, sample of inoculum was pipetted into the medium and incubated at various temperatures (20, 25, 30, 35 and 40 °C). Flocculating activity was then measured [32]. The culture medium was prepared based on previously obtained optimal growth conditions. The isolate was cultured at different shaking speeds in the range of 0–220 rpm for 72 h. Thereafter, the flocculating activity was determined as previously described [33]. The effect of incubation time on bioflocculant production was evaluated. The medium was composed based on previously obtained optimal growth conditions. The broth culture (2 mL) was withdrawn, centrifuged and evaluated for flocculating activity after every 12 h over a period of 120 h. The optical density (OD) of the broth was measured at 550 nm, representing bacterial biomass, and the pH was also recorded [34].

### 2.9. Extraction and Purification of the Bioflocculant

Extraction and purification of bioflocculant was done using the method of Dlamini et al. [35]. The bacterium was cultured under optimum culture conditions in 1 L of the medium and the broth culture was centrifuged (8000 rpm, at 4 °C for 30 min) to obtain the cell free supernatant. One volume of sterile distilled water was poured into the supernatant and re-centrifuged to remove insoluble materials. Thereafter, two volumes of alcohol were added to the supernatant; the mixture was agitated and then allowed to precipitate for 12 h at 4 °C. Then, the precipitate was vacuum dried, and the crude product was re-dissolved in the sterile distilled water to give a solution of 1% *w*/*v*. A mixture of chloroform and *n*-butyl alcohol (5:2 *v*/*v)* was added to the bioflocculant solution in a ratio of 2:1 (*v*/*v*). The mixture was agitated and was allowed to stand at room temperature for 12 h. The supernatant was collected; centrifuged (8000 rpm for 30 min at 4 °C) and dialyzed for 12 h against distilled water. The dialysate was vacuum-dried to obtain the purified bioflocculant.

### 2.10. Characterizations of the Bioflocculant

The elemental composition, functional groups and pyrolysis profile of the bioflocculant was investigated. Surface structure of bioflocculant was investigated using a scanning electron microscope (SEM) (SIGMA VP-03-67, ZEISS Microscopy, Cambridge, UK). Elemental analysis was carried out by an elemental detector (X-Max EDS System, Oxford Instruments Inc, Oxford, UK). The functional groups of the bioflocculant were evaluated using a Fourier transform infrared spectrophotometer (PerkinElmer UATR TWO, 2000, PerkinElmer LAS, Rodgau, Germany), with a spectral range of 500–4000 cm^−1^ [36]. The pyrolysis property of the bioflocculant was determined using of thermo-gravimetric analyser (Model: DTG-60, Shimadzu Corporation, Tokyo, Japan). The bioflocculant was heated in the range of 22 to 800 °C at a constant rate of 10 °C per minute under a constant flow of nitrogen gas [37].

### 2.11. Biosafety of the Bioflocculant

Biosafety of the bioflocculant was evaluated by determining its cytotoxicity on African green monkey kidney (Vero) and Bovine dermis using the 3-(4,5-dimethylthiazol-2-yl)-2,5-diphenyl tetrazolium bromide (MTT) assay. The cells were grown to a confluency of 80% in 25 cm^3^ flasks using complete culture medium (CCM: minimum essential medium (MEM) supplemented with 5% foetal calf serum and 0.1% gentamicin) and harvested. They were re-suspended in growth medium at a concentration of 5 × 10^4^ cells mL^−1^ and incubated for 24 h at 37 °C in 5% CO_2_ using a 96 well plate. Thereafter, the CCM was removed, and the cells were treated with the bioflocculant at different concentrations (50–200 µg/µL). Cells treated with 0.1% dimethyl sulfoxide (DMSO) served as the negative control and doxorubicin as the positive control. The cells were re-incubated at 37 °C in 5% CO_2_ for 24 h. After incubation, the medium was removed and supplemented with fresh CCM (100 µL). Thereafter, 30 µL of MTT reagent (5 mg/mL in phosphate buffered saline (PBS) was poured into the wells and incubated at 37 °C in 5% CO_2_ for 4 h. The MTT solution was aspirated from the wells and the formazan particles were dissolved in 50 µL of DMSO. MTT reduction was measured by reading the optical density (OD) of the samples at 540 nm using a micro plate reader (Varioskan Flash 3001, Thermo Fisher Scientific, Vantaa, Finland). Percentage cell inhibition (%CI) was assessed using the formula:

%CI = (A_o_ − A)/A_o_ × 100, (2)

where A_o_ and A represent the OD readings of untreated samples and treated samples, respectively, at 540 nm. The inhibitory concentration of 50% (IC_50_) values was calculated from the GraphPad Prism (V6.1) using linear regression method [38].

### 2.12. Effect Dosages, Cations and pH on the Flocculating Activity

The effect of bioflocculant concentrations of flocculating activity was determined according to Maliehe et al. [39]. Two milliliters of the different concentrations in the range of 0.2–1.0 mg/mL (*w*/*v*) were used. Thereafter, the flocculating activity was evaluated as previously stated. The method of Tsilo et al. [40] was used to determine the influence of cations on flocculation. Metal ions included 1% of NaCl, LiCl, KCl, MnCl_2_, MgCl_2_ and FeCl_3_. The metal ions replaced the CaCl*_2_* solution (3 mL, 1%) that was previously used in the flocculation assay and flocculating activity was measured. The pH of the kaolin clay solution (4 g/L) was varied between 3–12 using 0.1 M HCl and 0.1 M NaCl. Thereafter, the obtained optimum concentration was poured into a mixture of kaolin solution and 1% of the cation. Afterwards, the flocculating activity was determined at each pH level as described previously [41].

### 2.13. Application of the Bioflocculant in Wastewater Treatment

In the application process, the pure bioflocculant was used to treat wastewater from the Erwat Wastewater Treatment Plant from East Rand, Gauteng, South Africa. The pH of the wastewater was adjusted to 7 using 0.1 M HCl and 0.1 M NaCl. The wastewater was then treated with the bioflocculant. Inorganic flocculants, such as aluminium sulphate and ferric chloride, served as controls. The removal efficiencies of the flocculants on chemical oxygen demand (COD), biological oxygen demand (BOD) and sulphur were measured using appropriate test kits following the manufacturers’ instructions. Removal efficiency (RE) was expressed in percentage as:

%RE = (C_o_ − C)/C_o_ × 100, (3)

whereby C_o_ and C represent the values obtained before and after treatment, respectively, of the wastewater by the flocculants [7].

### 2.14. Statistical Analysis

All experiments were performed in triplicate and data expressed as mean values. Standard deviations were calculated. A one-way analysis of variance (ANOVA) was conducted using Graph Pad Prism™ version 6, GraphPad Software, San Diego, CA, USA. A significance difference level of *p* ˂ 0.05 was considered statistically significant.

## 3. Results and Discussion

### 3.1. Physiochemical Parameters of Water

Geographic location and environmental conditions are important factors that influence the distribution and activity of marine bioflocculant-producing bacteria. Thus, measurement of physiochemical parameters is important when isolating microorganisms. The physiochemical parameters of seawater were measured using the HI 98194 PH/E/DO multi-parameter. The temperature was 23.24 ± 0.49 °C, pH 7.13 ± 0.05, TDS 23.55 ± 5.0 mg/L, DO 42.41 ± 5.5 mg/L, salinity (30.81 ± 7.14 ppt), pressure (759.56 ± 0.5 mmHg), and specific conductivity was 47.09 ± 10.1 mS/cm.

### 3.2. Isolation and Identification of the Bioflocculant-Producing Bacterium 

A total of 31 isolates were isolated on two media with 15 isolates obtained on marine agar medium and 16 isolates on R2A medium. Among the 31 isolates, nine demonstrated flocculating activities higher than 50%, with Isolate MTZ08 revealing the highest flocculating activity of 68% against kaolin particles in solution. MTZ08 appeared non-pigmented, shiny to milky white, with a convex shape, circular and smooth. The bacterium was selected and identified using 16S rRNA gene analysis. The bacterium revealed 99% homology to *Ochrobactrum oryzae* AB84113. The genus *Ochrobactrum* is comprised of Gram-negative bacteria belonging to alpha-proteobacteria and *Brucellaceae* family [42]. The literature shows its application in the bioremediation of polychlorinated biphenyls and petroleum hydrocarbon [43,44]. *Ochrobactrum* sp. DGVK1 has been effectively used to remove N,Ndimethylformamide from wastewater and hexadecane from soils [45,46]. *Ochrobactrum oryzae* has been used to degrade wheat straw for production of biofuel [47] and has also been utilised in the removal of nitrogen [48].

### 3.3. Optimisation of Medium Composition and Culture Conditions

**Figure 1:**
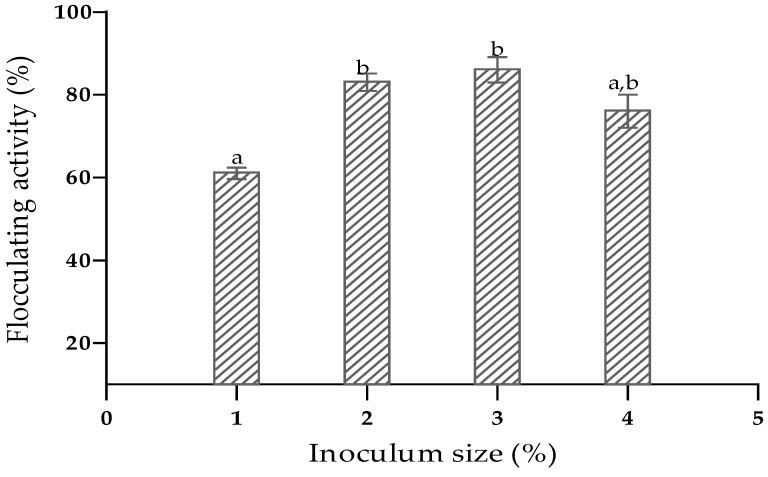
Effect of inoculum size on bioflocculant production. The letters (a,b) denote significant differences (*p* < 0.05).

The literature has previously highlighted the effects of medium composition and culture conditions on bioflocculant production [49]. To maximize bioflocculant yield by the isolate, the medium composition (carbon and nitrogen sources) and culture conditions (inoculum size, temperature, initial pH of the medium and cultivation time) were optimised. The volume of the bacterial inoculum inoculated into the medium has an important role in bioflocculant production. The maximum flocculating activity of 86% was obtained at an inoculum size of 3% (*v*/*v*). However, there was no significant difference (*p* > 0.05) between the activity of 86% by the inoculum size of 3% and the activity of 83% achieved by 2% (*v*/*v*) inoculum size (Figure 1). Hence, an inoculum size of 2% was preferred for the following experiments. The flocculating activity was low at 1% inoculum size due to the fact that the isolate might have had an extended lag phase, consequently delaying production of the bioflocculant. Moreover, an inoculum size of 4% might have caused excess overlapping of the bacterial niche, leading to inhibition of bioflocculant production. The literature shows that high flocculating activities are often obtained at low inoculum size ranging from 1 to 5%. Makapela et al. [50] recorded high bioflocculant yield at an inoculum size of 4%, whereas Luo et al. [28] reported optimum bioflocculant production at 1% inoculum.

Carbon source plays a vital role in the production of bioflocculants [51]. The effect of carbon sources on the bioflocculant by *O. oryzae* AB841138 was assessed, and the maximum flocculating activity of 92% was obtained when starch was used as the carbon source, followed by glucose (86%) and fructose (81 ± 9.1%). Sucrose yielded the least flocculating activity (43%) (Table 1). It was concluded that *O. oryzae* AB841138 was able to effectively assimilate starch for its growth energy and bioflocculant production. Likewise, *Klebsiella* sp. and *Sorangium cellulosum* had preference for starch for optimal bioflocculant production [27,52]. Therefore, in this study, starch was utilised for subsequent investigations.

Table 1 illustrates the effect of nitrogen sources on bioflocculant production by *O. oryzae* AB841138. All nitrogen sources enhanced bioflocculant production, yielding flocculating activities of 85% and above. The peak flocculating activity of 92% was obtained when yeast extract was used. This indicates that *O. oryzae* can use yeast extract to produce bioflocculant efficiently. Similar results were found by Deng et al. [53], who reported that *Bacillus mucilaginosus* is capable of utilising yeast extract to produce bioflocculant with good flocculating activity. Yeast extract was used as the nitrogen source in the following experiments in this study.

The effect of temperature on bioflocculant production by the selected isolate was determined. There was a significant increase in flocculating activity with an increase in the cultivation temperature from 20 to 30 °C. The highest flocculating activity of 93% was observed when the bacterium was grown at 30 °C, and the least activity of 77% obtained when the bacteria was cultured at 20 °C (Table 1). Profound flocculating activity implied that *O. oryzae* is a mesophile whose enzymatic reactions function effectively at 30 °C for biosynthesis of the bioflocculant. The significant (*p* < 0.05) decrease in flocculating activity observed at temperatures above 30 °C may be due to a decrease in enzyme activity resulting from denaturation. The low flocculating activities at low temperatures (<30 °C) might be due to slow bacterial growth rate, leading to poor production of the bioflocculant. Similar trends were noticed for *Klebsiella mobilis* by Wang et al. [54]. However, our observations are contrary to the findings of Giri et al. [55], who reported high yields when *Bacillus subtilis* F9 was cultivated at 40 °C. The results confirm that different bacterial strains prefer different temperatures for optimum bioflocculant production.

**Figure 2:**
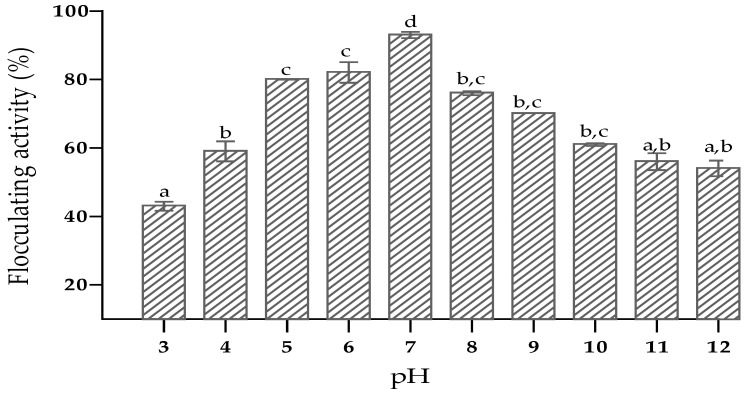
Effect of pH on bioflocculant production. The letters (a, b, c, d) denote significant differences (*p* < 0.05).

The relationship between initial pH of the culture medium and bioflocculant production by *O. oryzae* AB841138 was evaluated. The optimum initial pH of the medium was in the range of 5–7 with flocculating activities above 70%. The peak flocculating activity of 93% was observed at a pH of 7, while the lowest flocculating activity was obtained at a pH of 3. (Figure 2). The initial pH of the medium influences the electrical charges on bacteria strains and their oxidation–reduction potential, which, in turn, can affect nutrient absorption and enzyme activities within the bacterial cells, thus affecting bioflocculant production [56]. Therefore, it was concluded that *O. oryzae* AB841138 is a neutrophilic bacterium with the ability to produce bioflocculant effectively in the pH range of 5–7. *Nocardiopsis aegyptia* sp. nov and *Arthrobacter* sp. Raats are examples of netrophilics that revealed high flocculating activity when the initial pH of the media was adjusted to 7 [57,58]. 

**Figure 3:**
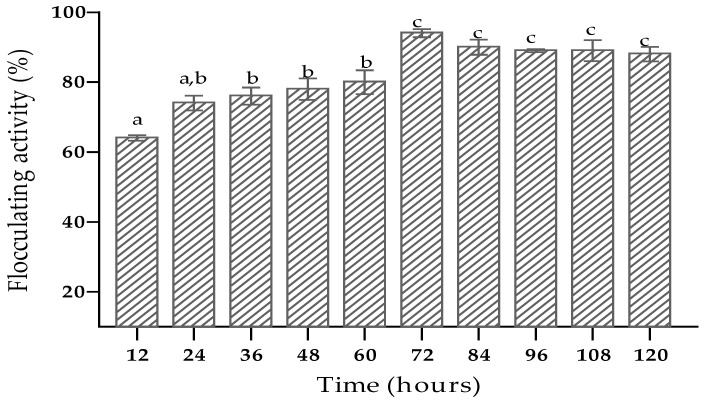
The impact of incubation time on bioflocculant production. The letters (a, b, c) denote significant differences (*p* < 0.05).

Different microorganisms prefer different incubation times for effective production of bioflocculants [58]. In this study, the flocculating activity increased with an increase in the incubation period up to 72 h. The flocculating activity was at a peak (94%) with an incubation time of 72 h (Figure 3). This meant that maximum bioflocculant production occurred during the late exponential stage or early stationary phase of growth. Similar findings have been obtained in other studies [59,60]. 

### 3.4. Extraction and Purification of Bioflocculant

The bioflocculant was obtained as a white powder after vacuum drying, and weighed 3.768 g. The bioflocculant yield was much higher than the 1.15 g/L recovered from *Bacillus firmus* [56] and the 1.36 g/L yield from *Proteus mirabilis* [22]. However, the yield was much lower than those reported by Natarajan [61], in which *Bacillus firmus* and *Bacillus licheniformis* produced 10 and 16.55 g/L. Therefore, there is a need to improve this strain to produce a higher yield.

### 3.5. Characterization of the Purified Bioflocculant

**Figure 4:**
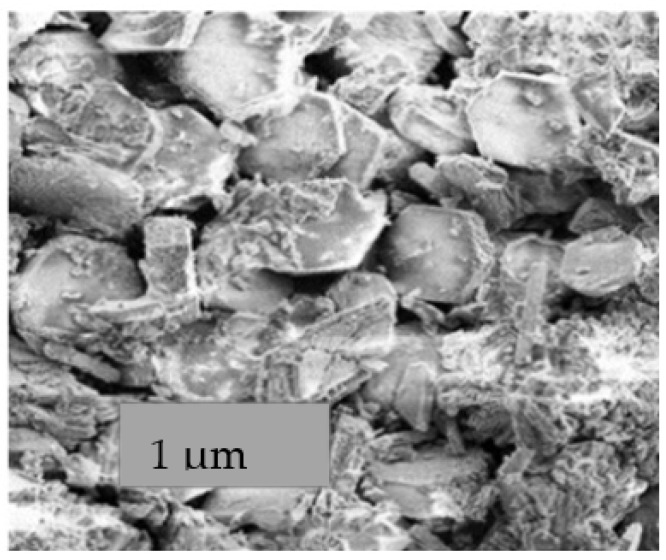
SEM image showing bioflocculant structure.

Figure 4 shows the surface structure of the bioflocculant. The bioflocculant had a hexagonal shape.

The elemental composition of the bioflocculant was investigated and the results are displayed in Table 2. The main elemental constituents were carbon (45.6%), oxygen (43.3%) and nitrogen (2.5%). Potassium and sulphur were present in the least amounts at 0.2%. The elemental composition of the bioflocculant was perceived to provide it with structural flexibility and stability [51]. The results show some similarities with the elemental composition of bioflocculant MBF-UFH from *Halomonas* sp. Okoh, i.e., carbon (17.21%), oxygen (40.04%) and nitrogen (6.66%) [62].

**Figure 5:**
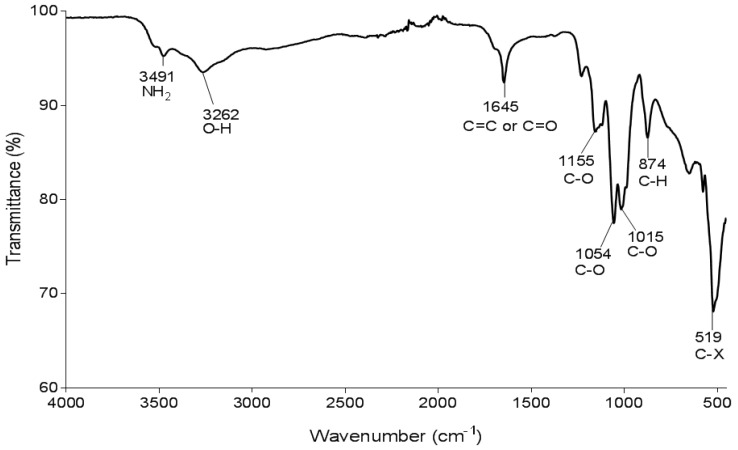
FTIR spectrum of the purified bioflocculant.

FTIR analysis was undertaken to ascertain and characterise the functional groups of the purified bioflocculant; the results are displayed in Figure 5. The FTIR spectrum showed a weak peak at 3491 cm^−1^ for an amino group (NH2), and a broad absorption peak at 3262 cm^−1^ representative of a hydroxyl group [63]. The absorption peak at 1645 cm^−1^ is due to the vibration of a carbonyl group in amide functionality [64]. The peaks between 1000–1200 cm^−1^ are due to the vibrations of an ester linkage and are generally characteristics of sugar derivatives [54]. The efficacy of bioflocculants strongly depends on their functional groups that serve as binding sites for colloidal particles in solutions [65]. Therefore, the observed functional groups enhance flocculation by serving as binding sites for kaolin particles. Moreover, the FTIR spectrum suggested the bioflocculant to possess polysaccharides and proteins. The observed results are comparable with findings from other studies [64,66,67].

**Figure 6:**
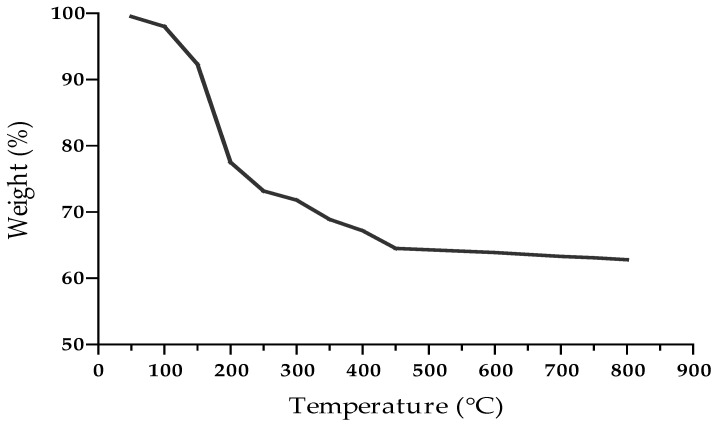
TGA spectrum of the bioflocculant from *O. oryzae* AB841138.

Thermogravimetric analysis (TGA) was used to determine the pyrolysis property of the bioflocculant. Figure 6 shows different profiles the bioflocculant. The bioflocculant lost 2% of its weight within the temperature range of 50 and 100 °C due to loss of moisture content [7]. When it was heated at 200 °C, the bioflocculant lost 22% of its total weight, and 35% at 450 °C. The weight loss at 200 and 460 °C could be related to degradation of the bioflocculant [60]. The bioflocculant maintained approximately 62.8% of its weight after being heated at 800 °C. The high yield obtained after being heated at 800 °C showed the thermal stability of the bioflocculant. The results in this study were similar to those of Maliehe et al. [39] in which the bioflocculant was found to be it thermally stable.

### 3.6. Biosafety of the Bioflocculant from O. oryzae AB841138

A tetrazolium-based calorimetric method was used to determine the cytotoxic effect of the bioflocculant on bovine dermis and African green monkey kidney (Vero) cells. The bioflocculant had a mean inhibition concentration (IC_50_) of 180 µg/mL against bovine dermis and > 500 µg/mL on African green monkey kidney cells. The toxicity threshold level for bioflocculants is considered significant when the IC_50_ < 30 µg/mL [68]. Therefore, the results suggested the bioflocculant to be non-cytotoxic. Moreover, the results affirmed the probable safe use of the bioflocculant in different applications. The results were in accordance with those obtained by Sharma et al. [69] in which the exopolymer from *Acinetobacter haemolyticus* showed no toxic effect on sheep blood cells. 

### 3.7. Effect of Dosage, Cations and pH on Flocculating Activity

**Figure 7:**
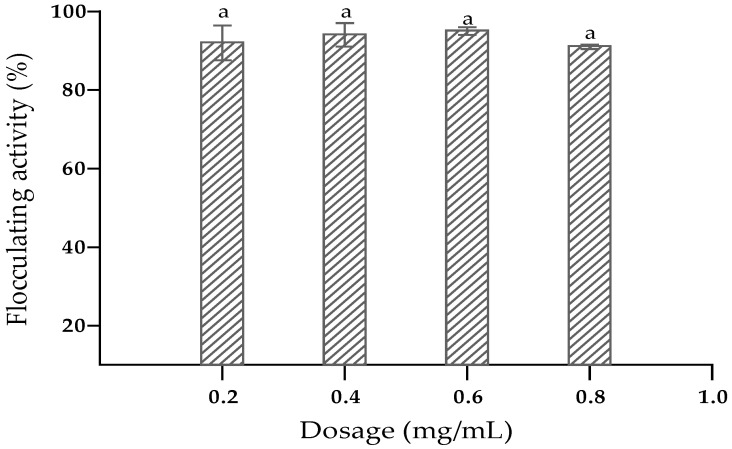
Impact of dosage size on flocculating activity. The letter (a) denotes insignificant differences (*p* > 0.05).

The effects of dosage size, cations and pH on the flocculating activity of the bioflocculant from *O. oryzae* AB841138 were evaluated. The bioflocculant achieved a maximum flocculating activity of 95% at a dosage size of 6 mg/mL. However, there was no statistical difference (*p* > 0.05) between the flocculating activity obtained at the dosage size of 0.6 mg/mL and that of 0.2 mg/mL, which achieved 92% activity (Figure 7). Therefore, the preferred dosage size was 0.2 mg/mL and was used in the following experiments. Dosage concentration is a crucial factor in the determination of the effective flocculation. Excess dosage concentration results in inhibition of the interaction between bioflocculant and kaolin clay in solution, leading to a decrease in flocculation [63]. Thus, the dosage size of 0.2 mg/mL was optimum in efficiently flocculating kaolin particles. These results are in agreement with those reported by Maliehe et al. [33].

The bioflocculant was capable of high flocculating activities of above 70% within a wide pH range of 3 to 12. At pH 5, the bioflocculant showed the highest activity of 90% (Table 3). The effectiveness of the bioflocculant under a wide pH range is economical and suitable for industrial applications [7]. The findings agree with those obtained by Zhang et al. [27], in which a bioflocculant from *Ruditapes philippinarum*, demonstrated high flocculating efficiency over a wide range of pH (pH 3–11). 

The impact of metal ions on flocculating activity of the bioflocculant was evaluated. Monovalent and divalent cations enhanced the flocculating efficiency of the bioflocculant, producing flocculating activities greater than or equal to 88%. The peak activity of 97% was observed when NaCl and MgCl were utilized. However, when a trivalent cation (Fe^3+^) was used, the flocculating activity drastically decreased (*p* < 0.05) (Table 3). Ferric chloride tends to lower the pH of water during its application as a flocculant [70]. Although the pH was adjusted to 7 prior to the application of Fe^3+^, the pH might have been lowered when Fe^3+^ was added, affecting bioflocculation. It should be noted that a high flocculating activity of 93% was observed in the absence of cations, indicating that the bioflocculant is cation independent, thus making it cost-effective. Similar results were reported for bioflocculants from *Klebsiella pneumoniae* and *Aspergillus flavus*, resulting in high flocculating activities in the absence of cations [71,72]. 

### 3.8. Removal Efficiency of the Bioflocculant

The bioflocculant and other flocculants (aluminium sulphate and ferric chloride) were compared in the treatment of wastewater from the Erwat Treatment Plant. The bioflocculant demonstrated 98% removal efficiency of COD, whereas aluminium sulphate and FeCl_3_ showed 97% and 98% efficiency, respectively (Table 4). Moreover, the bioflocculant effectively reduced BOD and sulphur concentrations by 91 and 86%, respectively. Aluminium sulphate had removal efficiencies of 82% and 90% for the removal of BOD and sulphur, while FeCl_3_ had 86% removal efficiency for BOD and 90% for sulphur. In general, the removal efficiencies of the bioflocculant were statistically similar to those observed when aluminium sulphate and FeCl_3_ were utilised in all parameters_._ This implies that the bioflocculant can be used as an alternative to chemical flocculants in wastewater treatment. Kaur et al. [73] presented similar results in which the bioflocculant effectively removed impurities from leachate.

## 4. Conclusions

The most promising bioflocculant-producing strain was identified as *O. oryzae* AB841138. The bioflocculant from *O. oryzae* AB841138 exhibited a maximum flocculating activity of 92% and a yield of 3.768 g/L when cultured optimally at pH 7, with an inoculum size of 1% (*v*/*v*) and when fructose and yeast extract were used as carbon and nitrogen sources, after cultivation at 30 °C for 72 h. The bioflocculant had a hexagonal structure with diverse functional groups such as hydroxyl, carboxyl, amine, and amide. The purified bioflocculant was found to be thermally stable and effective at a low dosage rate of 0.6 mg/mL. Moreover, the bioflocculant demonstrated negligible cytotoxicity on both bovine dermis and African green monkey kidney cells, indicative of its biosafety. It also had excellent removal efficiencies for tested pollutants in wastewater. These properties suggest potential applicability of the bioflocculant from *O. oryzae* AB841138 in wastewater treatment. For further studies involving cheaper substrates, the mode of action of the bioflocculant and its effectiveness in other wastewater effluents, should be undertaken.

## Figures and Tables

**Table 1:** Effect carbon and nitrogen sources and temperature on bioflocculant production.

Carbon Sources	FA(%) ± SD	Nitrogen Sources	FA(%) ± SD	Temperature (°C)	FA(%) ± SD
Sucrose	81 ± 2.1 ^a^	Casein	89 ± 0.5 ^a^	20	77 ± 0.1 ^a^
Molasses	70 ± 2.2 ^a^	Peptone	88 ± 2.2 ^a^	25	84 ± 0.2 ^b^
Lactose	66 ± 4.6 ^b^	Ammonia	91 ± 4.7 ^a^	30	93 ± 0.4 ^c^
Xylose	76 ± 1.2 ^a^	Urea	85 ± 3.2 ^a^	35	82 ± 0.6 ^b^
Sucrose	43 ± 0.6 ^c^	Yeast extract	92 ± 3.8 ^a^	40	80 ± 0.1 ^b^
Glucose	86 ± 7.1 ^a^				
Starch	92 ± 1.7 ^a^				

Flocculating activity, SD—Standard deviation FA with different letters (^a^, ^b^, ^c^) are significantly different (*p* < 0.05).

**Table 2:** Percentage elemental composition of the bioflocculant.

Element	% (*w*/*w*)
Potassium	0.2
Oxygen	43.3
Magnesium	1.7
Sodium	2.3
Sulphur	0.2
Nitrogen	2.5
Chlorine	1.3
Calcium	0.8
Carbon	45.6
Phosphorus	1.9

**Table 3:** Effect of pH and cations on flocculating activity.

pH	FA(%) ± SD	Cations	FA(%) ± SD
3	74 ± 0.2 ^a^	Control	93 ± 3.5 ^a^
4	79 ± 0.9 ^a^	Li^+^	88 ± 3.5 ^a^
5	90 ± 4.5 ^b^	Na^+^	97 ± 2.7 ^a^
6	89 ± 0.2 ^a^	K^+^	89 ± 3.8 ^a^
7	85 ± 0.4 ^b^	Ba^2+^	96 ± 4.4 ^a^
8	85 ± 1.0 ^b^	Mn^2+^	90±11.3 ^a^
9	84 ± 1.3 ^b^	Mg^2+^	97 ± 1.5 ^a^
10	81 ± 1.7 ^a,b^	Ca^2+^	96 ± 2.1 ^a^
11	80 ± 1.3 ^a,b^	Fe^3+^	47 ± 1.0 ^b^
12	75 ± 0.4 ^a^		

FA denotes flocculating activity, SD denotes standard deviation and the letters (^a^, ^b^) show statistical differences (*p* < 0.05).

**Table 4:** Removal efficiency of the bioflocculant from *O. oryzae* AB841138.

Flocculants	COD (%)	BOD (%)	Sulphur
Bioflocculant	98 ± 2.0 ^a^	91 ± 1.4 ^a^	86 ± 0.5 ^a^
Aluminium sulphate	97 ± 0.1 ^a^	82 ± 3.1 ^a^	90 ± 1.2 ^a^
FeCl_3_	98 ± 1.8 ^a^	86 ± 0.5 ^a^	90 ± 0.1 ^a^

The letter (^a^) illustrates statistical insignificance (*p* > 0.05).

## Data Availability

Not applicable.
